# A study on the relationship between recreational physical activity and audiovisual difficulty for older adults

**DOI:** 10.1038/s41598-024-55209-z

**Published:** 2024-03-25

**Authors:** Jipeng Zhang, Rui Feng, Yiwen Cao, Hongfei Mo

**Affiliations:** 1 School of Physical Education (Main Campus), Zhengzhou University, Zhengzhou, Henan People’s Republic of China; 2 College of Public Health, Zhengzhou University, Zhengzhou, Henan People’s Republic of China

**Keywords:** Older adults, Recreational physical activity, Hearing difficulty, Visual difficulty, Ageing, Diseases, Health care, Medical research, Risk factors

## Abstract

Audiovisual difficulty are especially common in older adults. Audiovisual difficulty seriously affect the quality of life of older adults in their later years. It is a top priority to find out the related factors, and to intervene and prevent them. The purpose of this study was to explore the relationship between recreational physical activities and audiovisual difficulty in older adults. We hope that older adults can reduce the risk of hearing and visual difficulty through scientific physical activity. A total of 4,886 people were sampled from the National Health and Nutrition Examination Survey (NHANES) from 2013 to 2018. Recreational physical activity was assessed through the Global Physical Activity Questionnaire (GPAQ); Hearing and visual difficulty were assessed using the Disability Questionnaire (DLQ). Chi-square test was used for categorical variables and rank sum test was used for measurement variables. P < 0.05 was considered statistically significant (bilateral test). After univariate analysis, binary Logistic regression analysis was performed with recreational physical activity as the independent variable, statistically significant demographic variable as the covariate, and hearing and visual difficulty as the dependent variable, respectively. (1) After excluding all confounding variables, recreational physical activity was significantly associated with hearing difficulty (P < 0.001), odds ratio (OR) 0.657 (95% CI 0.5899–0.733); (2) Recreational physical activity was significantly associated with visual difficulty (P < 0.001), OR 0.731 (95% CI 0.630–0.849). (1) Recreational physical activity is the protective factor of hearing difficulty in older adults; (2) Recreational physical activity is a protective factor for visual difficulty in older adults.

## Introduction

The world is facing the problem of population aging, which is an important medical and demographic problem for society. A study predicted that by 2050, there will be twice as many people aged 60 years or older than adolescents aged 10–24 years^1^. Due to physiological decline and aging of tissues and organs, older adults may inevitably suffer from some chronic diseases, such as diabetes, hypertension, coronary heart diase and hearing and visual difficulty. Among these chronic diseases, the incidence of audiovisual difficulty is high in the older adults, and hearing and visual difficulty will bring great troubles to the life of older adults.

As one of the health issues related to impaired sensory function, audiovisual difficulty affects people's physical and mental health in various ways. Hearing difficulty is very common in older adults and is the third most common chronic health condition among older adults^2^. It is estimated that by '44, there will be more than 20.2 million older adults with hearing difficulty^3^. Many aspects of daily life in older adults are related to hearing ability, and hearing difficulty can affect quality of life, social relationships, motor skills, psychological aspects, and the function and morphology of specific brain regions. Hearing difficulty can make people feel alone, helpless, and unresponsive, which in the long run can lead to social isolation, depression, and even dementia^4^. Visual difficulty has a similar effect. One study showed that 14.5% of 65–74 year olds in the U.S. suffer from visual difficulty, and 21.1% of those over the age of 75 suffer from visual difficulty^5^. Visual difficulty not only hinder people's daily life and social participation, but also lead to problems such as dependence, limited activities, and access to social institutions. In addition, it can also lead to an increase in mental and physical disabilities, which has a serious impact on the social function and quality of life of older adults^6,7^. The impact of audiovisual difficulty on people's body and mind is slow and long-term, and the impact is manifested in physiology, psychology and personality. Audiovisual difficulty are especially common among older adults, and China is one of the countries with the largest older adults population and the fastest growing aging rate in the world.

Audiovisual difficulty seriously affect the quality of life of older adults in their later years, and have become a serious social health problem. It is particularly important to find out the related factors and intervene and prevent them. Previous studies have shown that hearing and visual difficulty may be associated with lower levels of physical activity^8–11^. Different types of physical activities may have different associations with audiovisual abilities, and we hypothesized that recreational physical activity might be associated with a reduced probability of having audiovisual difficulty. Therefore, this study aims to explore the relationship between recreational physical activities and audiovisual difficulty in older adults, hoping that more and more older adults people can reduce the probability of developing audiovisual difficulty through scientific physical activities.

## Methods and materials

### Object

**Figure 1:**
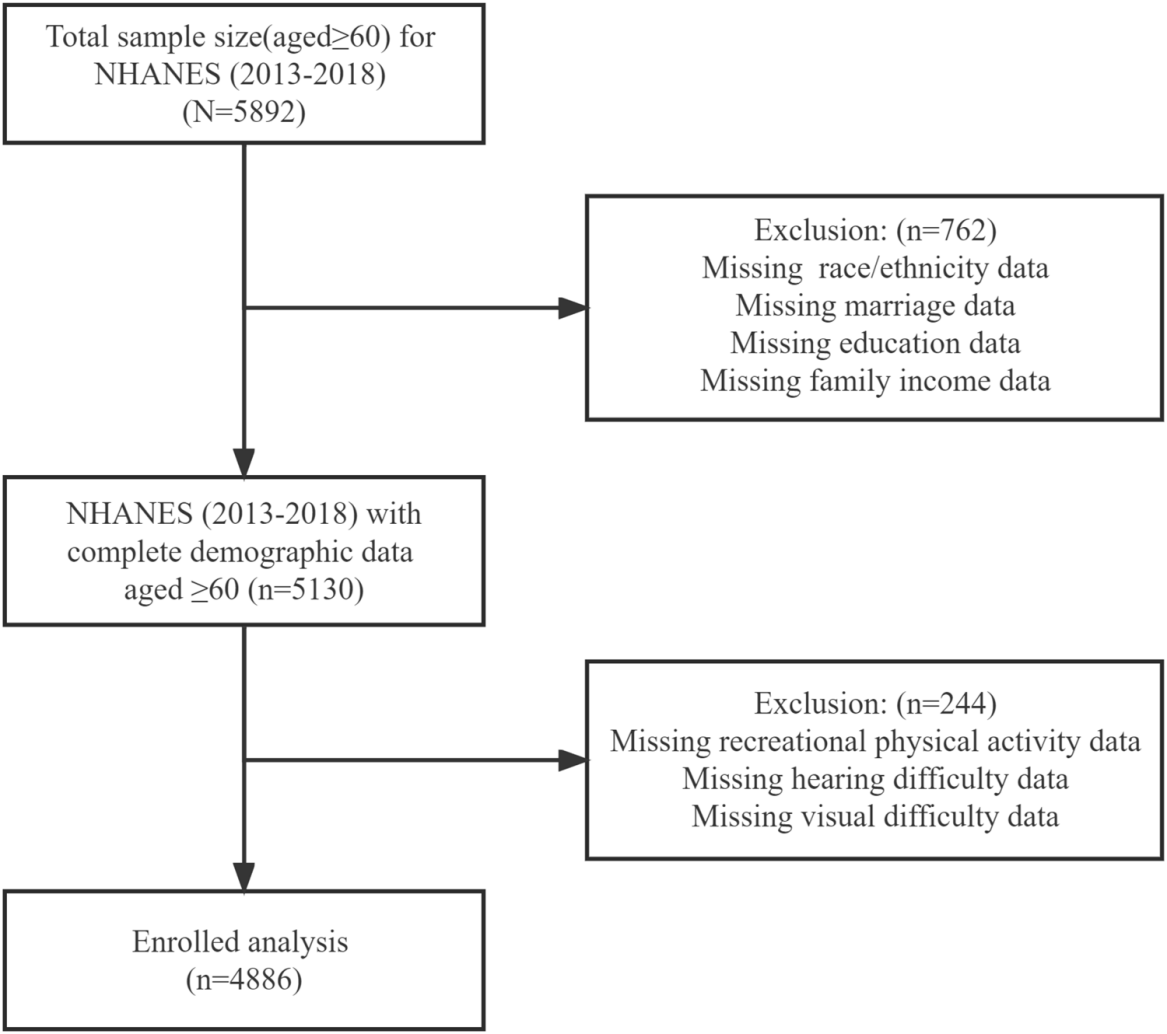
Data screening flow chart.

The NHANES is a population-based, cross-sectional survey designed to collect information on the health and nutrition status of the U.S. household population^12^. This study used a representative sample of 4,886 individuals aged 60 years and older stratified by NHANES from 2013 to 2018. NHANES covers about 15,000 households, all of which are U.S. residents who have lived in the United States for at least two months. The survey protocol and secondary analysis of the data were approved by the National Center for Health Statistics Ethics Review Committee, and all adult participants provided written notice of consent.^13^. Additional details on study design, sampling and exclusion criteria are shown in the figure below. (See Fig. 1).

### Covariate

Covariates included gender, age, race, education, marital status, and poverty ratio. Race is divided into Hispanic, non-Hispanic white, non-Hispanic black, non-Hispanic Asian, and other races. The education level is divided into below high school, high school and above high school. Marital status was divided into cohabitation, married living alone (widowed, divorced, separated) and never married. The income poverty ratio is calculated by dividing household (or individual) income by the poverty line specific to the year under survey. In this study, the poverty ratio was used to create two income conditions, poor (< 1.3) and middle income (≥ 1.3)^14^.

### Audiovisual difficulty assessment

Hearing and visual difficulty were assessed using the Disability Questionnaire (DLQ), which was asked at home by a trained interviewer using a computer-assisted Personal Interview (CAPI) system. In the DLQ, DLQ010 "Are you deaf, or do you have severe hearing difficulty?" The codes "1" and "2" represent hearing difficulty and no hearing difficulty, respectively. DLQ020 "Are you blind, or do you have trouble seeing even with glasses?" The codes "1" and "2" represent visual difficulty and no visual difficulty, respectively. Finally, the answer results of DLQ010 and DLQ020 were used to determine whether they had hearing difficulty and visual difficulty.

### Recreational physical activity assessment

In NHANES, The Physical Activity Questionnaire (PAQ) categorizes physical activity into three types: work-related physical activity, recreational physical activity, and commuting physical activity. The questionnaire assessed which type of physical activity the respondent engaged in during a typical week. Recreational physical activity data is extracted from the adult section of the (PAQ), which is based on the GPAQ, which provides interview data on respondent levels of physical activity. Participants 18 years of age and older are eligible for the adult portion of the PAQ. Questions are asked at home using the CAPI system by a trained interviewer. Determine whether the sample engaged in high- or moderate-intensity recreational physical activity in a typical week. The codes "1" and "2" indicate whether recreational physical activity is present.

### Statistical analysis

We used Microsoft Excel 2010 to extract and merge the raw data and exclude missing and useless (rejected, don't know) items. The database includes people 60 years of age and older with complete information. We examined the significance of differences in covariates between the "hearing difficulty" and "no hearing difficulty" groups and between the "visual difficulty" and "no visual difficulty" groups. Rank sum test was used for continuous variables and chi-square test for categorical variables. We used a binary Logistic regression model to analyze the relationship between hearing and visual difficulty and recreational physical activity, respectively. All data were analyzed using Statistical Product and Service Solutions (SPSS) version 26.0, and a P-value less than 0.05 was considered statistically significant (bilateral test). Variables that were statistically significant in the univariate analysis were included in the stepwise binary Logistic regression analysis. A-entry = 0.05 and a-exit = 0.10 were used to select and exclude confounding variables.

When hearing difficulty was taken as the dependent variable and recreational physical activity as the independent variable, all variables (P < 0.05) were statistically significant in univariate analysis except income poverty ratio (P = 0.230). In the significance test of measurement data, age P = 0.01 (variance not homogeneous), P (bilateral) < 0.001, the difference was not statistically significant. When analyzing the relationship between recreational physical activity and hearing difficulty, we took recreational physical activity as the independent variable (1 = yes, 2 = no) and hearing difficulty (1 = no hearing difficulty, 2 = no hearing difficulty) as the dependent variable. To exclude the effect of covariates, we built the following models: Model I: Only the independent variable recreational physical activity was adjusted; Model II: Adjusted for independent variables in Model I plus demographic variables (gender, race, education, and marital status); Model III: Adjusted for Model II plus the variable of visual difficulty.

When visual difficulty was the dependent variable and recreational physical activity was the independent variable, all the other variables (P < 0.05) were statistically significant in univariate analysis except gender (P = 0.325) and marital status (P = 0.341). In the significance test of measurement data, age P = 0.014 (variance not homogeneous), P (bilateral) = 0.002, the difference was not statistically significant. When analyzing the relationship between recreational physical activity and visual difficulty, we took recreational physical activity as the independent variable (1 = yes, 2 = no) and visual difficulty (1 = no visual difficulty, 2 = no visual difficulty) as the dependent variable. To exclude the effect of covariates, we built the following models: Model IV: Only the independent variable recreational physical activity was adjusted; Model V: Adjusted for independent variables in Model IV plus demographic variables (race, education, and income-poverty ratio); Model VI: Adjusted for model VI plus variables for hearing difficulty.

### Ethics approval and consent to participate

All procedures performed in the study were in accordance with the Declaration of Helsinki. The study protocols for NHANES were approved by the National Center for Health Statistics (NCHS) Research Ethics Review Board (Protocol#2017-1). All adult participants provided written notification of consent before participating in the study.

## Results

### Demographic characteristics

The study included a total of 4,886 adults aged 60 years and above in the 2013–2018 U.S. National Nutrition Examination Survey cycle who completed data on recreational physical activity levels, hearing difficulty, visual difficulty, and other demographic information.

There were significant differences in gender (P < 0.001), age (P < 0.001), race (P < 0.001), education level (P < 0.001), marital status (P = 0.005), recreational physical activity (P < 0.001) and visual difficulty (P < 0.001) between the group with hearing difficulty and the group without hearing difficulty. No difference was observed in the income-poverty ratio (P = 0.230). There were significant differences in age (P < 0.001), race (P < 0.001), education level (P < 0.001), income poverty ratio (P < 0.001), recreational physical activity (P < 0.001) and hearing difficulty (P < 0.001) between the group with visual difficulty and the group without visual difficulty. No differences were observed in gender (P = 0.325) and marital status (P = 0.341). (See Table 1).

**Table 1:** Demographic characteristics of older adults aged 60 years and above with audiovisual difficulty.

Characteristics, n%	Sample Capacity	Hearing difficulty	No	Test statistics	P	Visual difficulty	No	Test statistics	P
	N = 4886	n = 866	n = 4020			n = 506	n = 4380		
Gender				54.896^a^	< 0.001***			0.969^a^	0.325
Male	2438 (49.90)	531 (61.32)	1907 (47.44)			242 (47.83)	2196 (51.05)		
Female	2448 (50.10)	335 (38.68)	2113 (52.56)			264 (52.17)	2184 (48.95)		
Age	70.01 ± 6.92	72.96 ± 6.96	69.37 ± 6.73	− 13.418^b^	< 0.001***	70.93 ± 7.16	69.90 ± 6.88	− 3.115^b^	0.002**
Race				132.399^a^	< 0.001***			47.668^a^	< 0.001***
Hispanic	1106 (22.64)	201 (23.21)	905 (22.51)			172 (33.99)	934 (22.64)		
Non-Hispanic White	2196 (44.94)	507 (58.55)	1689 (42.01)			192 (37.94)	2004 (33.71)		
Non-Hispanic Black	1016 (20.79)	84 (9.70)	932 (23.18)			99 (19.57)	917 (23.18)		
Non-Hispanic Asian	439 (8.98)	41 (4.73)	398 (9.90)			27 (5.34)	412 (15.72)		
Other	129 (2.65)	33 (3.81)	96 (2.40)			16 (3.16)	113 (4.75)		
Education				16.120^a^	< 0.001***			89.417^a^	< 0.001***
Below high school	1302 (26.65)	274 (31.64)	1028 (25.57)			221 (43.68)	1081 (17.96)		
High school	1153 (23.60)	208 (24.02)	945 (23.51)			112 (22.13)	1041 (24.30)		
Post high school	2431 (49.75)	384 (44.34)	2047 (50.92)			173 (34.19)	2258 (57.74)		
Marital Statues				10.620^a^	0.005**			2.149^a^	0.341
Cohabitation	2766 (56.61)	484 (55.89)	2282 (56.77)			271 (53.56)	2495 (61.78)		
Married living alone	1855 (37.97)	353 (40.76)	1502 (37.36)			206 (40.71)	1649 (20.33)		
Not married	265 (5.42)	29 (3.35)	236 (5.87)			29 (5.73)	236 (17.89)		
Income to Poverty				1.441^a^	0.230			73.849^a^	< 0.001***
Impoverished	1502 (30.74)	281 (32.45)	1221 (30.37)			240 (47.43)	1262 (27.12)		
Moderate income	3384 (69.26)	585 (67.55)	2799 (69.63)			266 (52.57)	3118 (72.88)		
Physical Activity				34.437^a^	< 0.001***			27.842^a^	< 0.001***
Yes	1880 (38.48)	257 (29.68)	1623 (40.37)			139 (27.47)	1724 (47.08)		
No	3006 (61.52)	609 (70.32)	2397 (59.63)			367 (72.53)	2639 (52.92)		
Visual difficulty				161.372^a^	< 0.001***				
Yes	506 (10.36)	193 (22.29)	313 (7.79)						
No	4380 (89.64)	673 (77.71)	3707 (92.21)						
Heating difficulty								161.372^a^	< 0.001***
Yes						193 (38.14)	673 (15.37)		
No						313 (61.86)	3707 (84.63)		

*P < 0.05, **P < 0.01, ***P < 0.001, Figures in parentheses are percentages.

^a^Chi-square test.

^b^Rank sum test.

### Relationship between hearing difficulty and recreational physical activities

In Logistic regression analysis, model I (without excluding any covariates) showed that recreational physical activity was associated with hearing difficulty with an odds ratio (OR) of 0.630 (95% CI 0.567–0.700). Model II (excluding demographic variables) shows that OR 0.634(95% CI 0.569–0.706). Model III (excluding all covariates) shows OR 0.657(95% CI 0.5899–0.733). The study results indicated that engaging in recreational physical activity was associated with a decreased likelihood of developing hearing difficulty, after adjusting for all covariates. The probability of developing hearing difficulty for people with recreational physical activity is 65.7% of that for people without recreational physical activity. (P < 0.01) (See Table 2).

**Table 2:** Logistic regression analysis of recreational physical activity and hearing difficulty.

Mode	B	SE	Wald	P	OR (95% CI)
I^a^	− 0.462	0.054	73.797	< 0.001***	0.630 (0.567–0.700)
II^b^	− 0.456	0.055	68.225	< 0.001***	0.634 (0.569–0.706)
III^c^	− 0.420	0.056	56.409	< 0.001***	**0.657 (0.589–0.733)**

***P < 0.001.

^a^Only the independent variable recreational physical activity was adjusted.

^b^Adjustments were made for independent variables in Model I plus demographic variables (gender, race, education, and marital status).

^c^Adjustments were made according to Model II plus the variable of visual difficulty.

### Relationship between visual difficulty and recreational physical activities

In Logistic regression analysis, Model IV (without excluding any covariates) showed an odds ratio (OR) = 0.569 (95% CI 0.493 to 0.656) for the association between recreational physical activity and visual difficulty. Model V (excluding demographic variables) shows that OR 0.671 (95% CI 0.580–0.777); Model VI (excluding all covariates) shows that OR 0.731 (95% CI 0.630–0.849). The study results indicated that engaging in recreational physical activity was associated with a decreased likelihood of developing visual difficulties, after adjusting for all covariates. The probability of developing visual difficulty for those with recreational physical activity was 73.1% of that for those without recreational physical activity. (P < 0.01) (See Table 3).

**Table 3:** Logistic regression analysis of recreational physical activity and visual difficulty.

Mode	b	SE	Wald	P	OR (95% CI)
IV^a^	− 0.564	0.073	60.073	< 0.001***	0.569(0.493–0.656)
V^b^	− 0.399	0.075	28.531	< 0.001***	0.671(0.580–0.777)
VI^c^	− 0.313	0.076	17.003	< 0.001***	0.731(0.630–0.849)

***P < 0.001.

^a^Only the independent variable recreational physical activity was adjusted.

^b^Adjustments were made for independent variables in Model IV plus demographic variables (race, education, and income-poverty ratio).

^c^Adjustments were made according to model V plus the variable of hearing difficulty.

## Discussion

Using Logistic regression analysis of NHANES data from 2013 to 2018, we found that recreational physical activity was independently associated with both hearing and visual difficulty in people over 60 years of age. Recreational physical activity reduces the risk of hearing and visual difficulty, the risk of hearing difficulty in older adults with recreational physical activity is reduced by 26.70–41.10%, and the risk of visual difficulty in older adults with recreational physical activity is reduced by 15.1–37.0%. The findings come from an independent analysis of the recreational physical activity variable. Therefore, we will discuss "hearing difficulty and recreational physical activity in older adults" and "visual difficulty and recreational physical activity in older adults" respectively in the following texts.

### Hearing difficulty and recreational physical activity in older adults

Our findings are similar to those of previous studies in that adequate physical activity can effectively reduce the degree of hearing difficulty^15,16^. Related studies have shown that a slower pace is independently associated with hearing difficulty^17,18^, that is, an appropriate increase in physical activity can reduce the risk of hearing difficulty. In Spanish men, physical inactivity and obesity were significantly associated with audiovisual difficulty^19^, with an increase in physical activity and a decrease in the degree of audiovisual difficulty. More dispersed physical activity patterns in older adults are one of the important factors contributing to hearing difficulty^20^, which may be related to a lack of sustained physical activity in older adults. Among the physiological indicators, hearing difficulty may be related to blood pressure and respiratory rate^21^, because physical activity can improve blood pressure and slow down respiratory rate, and when blood pressure and respiratory rate are improved, the degree or likelihood of hearing difficulty in older adults is reduced. In addition, adolescents with less physical activity and more sedentary time tend to have more deaf-mute problems than their peers^22^, which also reflects that physical activity is a protective factor for hearing difficulty.

Our research conclusion may be explained by the following mechanisms: Firstly, recreational physical activity is associated with a lower risk of hearing difficulty, possibly because the central auditory system can be affected by exercise, and possibly because physical activity can improve the peripheral auditory processing ability of cochlea and improve the comprehension of auditory information^23^. Secondly, Physical activity promotes health and muscle growth, and higher muscle mass and performance health is associated with a lower incidence of hearing loss^15^. The participation of older adults in recreational physical activities can slow down the decline of many organs and systems in the body^24,25^. Studies have shown that impaired lower limb function, frailty syndrome, and disability may lead to varying degrees of hearing difficulty^26^. We believe that through physical activity, the decline of limbs, organs and body systems in older adults is slowed down, and the risk of hearing difficulty is correspondingly reduced. Thirdly, recreational physical activity can effectively reduce the risk of depression and cognitive decline in the elderly^27^. Mental health and cognitive balance are important factors in reducing physical diseases, and the risk of hearing difficulty in old adults will also be reduced. Finally, increased recreational physical activity is associated with vestibular function recovery^28^, which may directly lead to a reduced risk of hearing difficulty.

### Visual difficulty and recreational physical activities in older adults

Studies have shown that lower levels of physical activity are associated with multiple eye diseases, including glaucoma, age-related macular degeneration, and diabetic retinopathy^11^. In older adults, there was a significant decline in five of the six categories of physical activity, all with visual difficulty^29^. In American adults, walking and physical activity are associated with visual difficulty^30^. In a study of Irish people, physical activity in older adults was associated with better visual conditions^31^.

The relationship between recreational physical activity and vision may have the following mechanisms: Firstly, recreational physical activity can effectively prevent depression, cognitive decline and other symptoms^32^. Negative emotion is one of the factors that lead to visual difficulty, therefore, recreational physical activity can be a protective factor for visual difficulty. Secondly, through appropriate recreational physical activities, the field of vision can be larger, the recognition field of vision is larger, the motion perception field is larger, the hidden slope of far and far is lower, and the vision is more consistent, the depth perception is more accurate, the dynamic vision is better, and the eye movement is better. For example, athletes have better visual abilities than non-athletes, and better athletes have better visual abilities than poor athletes^33^. Thirdly, scientific physical activity can improve physical and mental health, including reducing the risk of all-cause death, chronic disease and premature death, and improving muscle strength, cardiorespiratory function and blood circulation. Older adults are as active as their abilities and conditions allow, and for substantial health benefits, older adults perform weekly aerobic, muscle strengthening and stretching exercises, as well as balance exercises as needed^34^ to prevent a variety of physical health problems, including vision difficulty in older adults. Our results support the conclusions of previous studies.

### Limitations of this study

This study has the following limitations: (1) We are not perfect in excluding confounding factors: The etiology of audiovisual difficulty is multi-factorial, including a variety of genetic, biological, environmental and social factors, and this study could not exclude all influencing factors. (2) The sample size of this study is limited, which may lead to the accuracy of data analysis and insufficient representation of samples. (3) Due to the limitation of NHANES, we could not obtain the specific extent of participants' hearing or vision difficulty, which limited our results to some extent.

## Conclusions

Recreational physical activity is a common protective factor for hearing and visual difficulty. It should be emphasized that this study is recreational physical activity, and different types of physical activity may have different effects on hearing and vision. In addition, audiovisual and physical activity are mutually reinforcing and promoting each other.

## Data Availability

The datasets generated and/or analysed during the current study are available in the [NHANES] repository, [NHANES Questionnaires, Datasets, and Related Documentation (cdc.gov)]. Raw data supporting the obtained results are available at the corresponding author.

## Abbreviations

NHANES: National Health and Nutritional Examination Survey
SPSS: Statistical Product and Service Solutions
GPAQ: Global Physical Activity Questionnaire
DLQ: Disability Questionnaire
PAQ: Physical Activity Questionnaire
CAPI: Computer-assisted personal interviewing
OR: Odds ratio
CI: Confidence interval
P: P-value
